# Development and validation of a prediction model for in-hospital death in patients with heart failure and atrial fibrillation

**DOI:** 10.1186/s12872-023-03521-3

**Published:** 2023-10-11

**Authors:** Meiyu Yan, Huizhu Liu, Qunfeng Xu, Shushu Yu, Ke Tang, Yun Xie

**Affiliations:** Department of Cardiology, Putuo People’s Hospital Affiliated to Tongji University, 1291# Jiangning Road, Putuo District, Shanghai, 200060 China

**Keywords:** Heart failure, Atrial fibrillation, Prediction model, In-hospital mortality

## Abstract

**Background:**

To develop a prediction model for in-hospital mortality of patients with heart failure (HF) and atrial fibrillation (AF).

**Methods:**

This cohort study extracted the data of 10,236 patients with HF and AF upon intensive care unit (ICU) from the Medical Information Mart for Intensive Care (MIMIC). The subjects from MIMIC-IV were divided into the training set to construct the prediction model, and the testing set to verify the performance of the model. The samples from MIMIC-III database and eICU-CRD were included as the internal and external validation set to further validate the predictive value of the model, respectively. Univariate and multivariable Logistic regression analyses were used to explore predictors for in-hospital death in patients with HF and AF. The receiver operator characteristic (ROC), calibration curves and the decision curve analysis (DCA) curves were plotted to evaluate the predictive values of the model.

**Results:**

The mean survival time of participants from MIMIC-III was 11.29 ± 10.05 days and the mean survival time of participants from MIMIC-IV was 10.56 ± 9.19 days. Simplified acute physiology score (SAPSII), red blood cell distribution width (RDW), beta-blocker, race, respiratory rate, urine output, coronary artery bypass grafting (CABG), Charlson comorbidity index, renal replacement therapies (RRT), antiarrhythmic, age, and anticoagulation were predictors finally included in the prediction model. The AUC of our prediction model was 0.810 (95%CI: 0.791–0.828) in the training set, 0.757 (95%CI: 0.729–0.786) in the testing set, 0.792 (95%CI: 0.774–0.810) in the internal validation set, and 0.724 (95%CI: 0.687–0.762) in the external validation set. The calibration curves of revealed that the predictive probabilities of our model for the in-hospital death in patients with HF and AF deviated slightly from the ideal model. The DCA curves revealed that the use of our prediction model increased the net benefit than use no model.

**Conclusion:**

The prediction model had good discriminative ability, and might provide a tool to timely identify patients with HF complicated with AF who were at high risk of in-hospital mortality.

**Supplementary Information:**

The online version contains supplementary material available at 10.1186/s12872-023-03521-3.

## Background

Atrial fibrillation (AF) is a prevalent clinical arrhythmia, and AF and heart failure (HF) are common co-existing diseases [1]. More than one-third of newly diagnosed AF patients have HF, and more than half of newly diagnosed HF patients have AF [2]. The presence of HF and AF significantly contributed to cardiovascular morbidity and mortality in the general population, and portends worse outcomes [3]. Compared with patients with AF or HF, patients with both have a higher risk of death [4]. A previous meta-analysis data of more than 50 000 patients demonstrated that in patients with HF, AF is associated with 40% higher odds of death among patients included in randomized trials and 14% higher odds of death in patients in observational studies [5]. Early identification of HF and AF patients with high mortality risk is of great significance for the implementation of medical decision-making and the reduction of disease burden.

Previously, factors such as catheter ablation, drug treatments, and red blood cell distribution width (RDW) were reported to be associated with the risk of mortality of HF patients or AF patients [6–8]. Several scoring systems or models have been published for the prediction of mortality in HF patients [9–11]. A risk score for in-hospital mortality in patients hospitalized with HF using American Heart Association Get With the Guidelines-Heart Failure (GWTG-HF) program data was also identified and widely applied [12]. A meta-analysis revealed that the prediction effect of the existing model was mediocre, with an average C-index of about 0.66, and the included population was mainly from strictly screened randomized controlled trials or medical claim data, which had limited extension possibility to other populations [13, 14]. In addition, these prediction models mainly focus on HF patients, and few studies have constructed prediction models for the risk of mortality of HF patients with AF.

MIMIC-III database is a large open-access database comprising deidentified health-related data associated with over forty thousand patients who stayed in critical care units of the Beth Israel Deaconess Medical Center between 2001 and 2012. The database included information such as demographics, vital sign measurements made at the bedside, laboratory test results, procedures, medications, caregiver notes, imaging reports, and mortality (https://mimic.mit.edu/docs/iii/) [15]. MIMIC-IV database, constructed based on MIMIC-III, and incorporated numerous improvements over MIMIC-III (https://mimic.mit.edu/docs/iv/) [16]. MIMIC-IV contains over 70,000 ICU admissions across the United States collected from 2008 to 2019 including comprehensive patient information. The eICU Collaborative Research Database (eICU-CRD) is a multicenter database including more than 200,000 ICU admissions in the United States [17].

In view of the co-morbidification burden of HF and AF, this study intended to develop a prediction model for in-hospital mortality of HF patients with AF based on the data from the Medical Information Mart for Intensive Care (MIMIC)-III and MIMIC-IV database. And the data from eICU-CRD were used as an external validation set. The prediction performance of the prediction model was evaluated and compared with GWTG-HF risk score.

## Methods

### Study design and population

In total, this cohort study extracted the data of 13,183 patients diagnosed as HF with AF upon intensive care unit (ICU) admission who aged ≥ 18 years old from the MIMIC-III (*n* = 4679), MIMIC-IV database (*n* = 7097) and eICU-CRD (*n* = 1407). HF and AF were diagnosed based on the International Classification of Disease (IC) codes. HF were diagnosed according to ICD-9 (42,821, 42,822, 42,823, 42,831, 42,832, 42,833, 42,841, 42,842, and 42,843), and ICD-10 (I5021, I5022, I5023, I5031, I5032, I5033, I5041, I5042, I5043, I50811, I50812, and I50813). AF was diagnosed according to ICD-9 (42,731), and ICD-10 (I480, I481, I482, and I4891). MIMIC-III (Medical Information Mart for Intensive Care III) is a large, freely-available database comprising deidentified health-related data associated with over forty thousand patients who stayed in critical care units of the Beth Israel Deaconess Medical Center between 2001 and 2012, including information such as demographics, vital sign measurements made at the bedside (~ 1 data point per hour), laboratory test results, procedures, medications, caregiver notes, imaging reports, and mortality (https://mimic.mit.edu/docs/iii/) [15]. MIMIC-IV constructed based on MIMIC-III, and incorporated numerous improvements over MIMIC-III (https://mimic.mit.edu/docs/iv/) [16]. The eICU-CRD is publicly available database comprising de-identified health data associated with more than 200,000 admissions to ICUs across the United States between 2014 and 2015 (https://eICU-crd.mit.edu/about/eICU/) [18]. In our study, those without survival information and hospitalized in the ICU < 24 h were excluded. Finally, 11,455 patients were included [MIMIC-III (*n* = 4238), MIMIC-IV (*n* = 5998), and eICU-CRD (*n* = 1219)]. The requirement of ethical approval for this was waived by the Institutional Review Board of Putuo People’s Hospital affiliated to Tongji University, because the data was accessed from MIMIC (a publicly available database). The need for written informed consent was waived by the Institutional Review Board of Putuo People’s Hospital affiliated to Tongji University due to retrospective nature of the study. All methods were performed in accordance with the relevant guidelines and regulations.

### Potential predictors

Demographic data including age (years), weight (kg), gender, race (Black, White, other or unknown), insurance (Medicare or other), and marital status (divorced, married, single, or widowed), laboratory data including heart rate (bpm), systolic blood pressure (mmHg), diastolic (mmHg), respiratory rate (bpm), temperature (℃), oxygen saturation (SpO_2_) (%), Charlson comorbidity index, the simplified acute physiology score (SAPS)-II, Glasgow coma scale (GCS), white blood cells (WBC) (K/uL), platelets (K/uL), hemoglobin (g/dL), RDW (%), creatinine (mg/dL), international normalized ratio (INR), prothrombin time (PT) (sec), partial thromboplastin time (PTT) (sec), blood urea nitrogen (BUN) (mg/dL), Glucose (mg/dL), anion gap (mEq/L), urine output (mL), and sodium (mEq/L), treatments during 24-h ICU admission including ventilation (no or yes), vasopressor (no or yes), renal replacement therapies (RRT) (no or yes), coronary artery bypass grafting (CABG) (no or yes), catheter (no or yes), antiarrhythmic (no or yes), antiplatelet (no or yes), anticoagulation (no or yes), beta-blocker (no or yes), and diuretic (no or yes), and other data including chronic obstructive pulmonary disease (COPD) (no or yes) first care unit [coronary care unit (CCU), cardiac vascular ICU (CVICU), medical ICU (MICU), surgical ICU (SICU) or other] were potential predictors analyzed in this study. All the data were collected within 24 h on admission to ICU, and the first measurement on ICU admission was applied for the prediction model construction.

### Outcome variable

The outcome in this study was the mortality 24 h into the ICU visit until the hospital discharge, which was defined as mortality status from 24-h admission to the ICU to hospital discharge. The beginning of follow-up was considered 24 h of the patient’s ICU admission. The date of death was obtained from the US government’s Social Security Death Index records and should not exceed the discharge date from the hospital. The mean survival time of participants from MIMIC-III was 11.29 ± 10.05 days and the mean survival time of participants from MIMIC-IV was 10.56 ± 9.19 days.

### Statistical analysis

Mean ± standard deviation (SD) was used to describe the measurement data subject to normal distribution, and t-test was used to compare the difference between the two groups. Medians and quartiles [M (Q_1_, Q_3_)] were employed to display the measurement data with abnormal distribution. Wilcoxon rank sum test was used to compare the difference between the two groups. Enumeration data were expressed as number of cases and percentages [n (%)], and differences between groups were compared using Chi-square test or Fisher’s exact probability method. The subjects from MIMIC-IV were divided into the training set to construct the prediction model, and the testing set to verify the performance of the model. The samples from MIMIC-III database were included as the internal validation set and the samples from eICU-CRD were included as the external validation set to validate the predictive value of the model. Univariate and multivariable Logistic regression analyses were used to explore predictors for in-hospital death in patients with HF and AF. The odd ratios (OR) and 95% confidence interval (95%CI) were applied as effect size. The receiver operator characteristic (ROC), calibration curves and the decision curve analysis (DCA) curves were plotted to evaluate the predictive values of the model. The area under the curve (AUC), sensitivity, specificity, negative predictive value (NPV), positive predictive value (PPV) and accuracy of the models for predicting the risk of in-hospital death in patients with HF and AF were measured. The confidence level alpha = 0.05. Data analysis, ROC curve plotting, difference comparison, construction of the prediction model, and Delong test were completed by SAS 9.4 (SAS Institute Inc., Cary, NC, USA). Visualization of nomogram and DCA curves were done by R version 4.2.1 (2022–06-23 ucrt). *P* < 0.05 was considered statistically significance.

## Results

### Comparisons between the characteristics of subjects in the survival group and death group

**Fig. 1:**
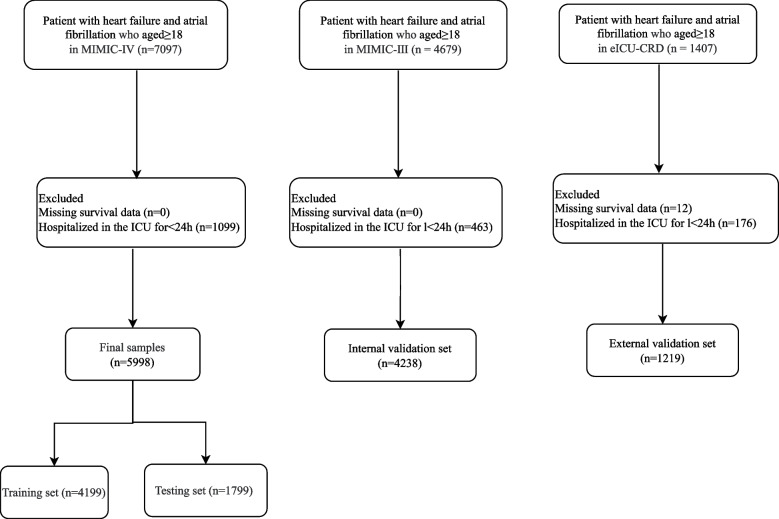
The screen process of participants

In our study, 13,183 patients with HF and AF who aged ≥ 18 years old from the MIMIC-III (*n* = 4679), MIMIC-IV database (*n* = 7097) and eICU-CRD (*n* = 1407) were included. There were 12 people lost survival information in eICU-CRD. In total, 463 subjects from MIMIC-III database, 1099 participants from MIMIC-IV database and 176 patients from eICU-CRD who hospitalized in the ICU < 24 h were excluded. Finally, 11,455 patients were included with 4238 from MIMIC-III, 5998 from MIMIC-IV and 1219 from eICU-CRD. The screen process was presented in Fig. 1.

In the samples from MIMIC-III database, there were 680 (16.05%) participants died at the end of the follow-up. The percentages of participants receiving ventilation (51.85% vs 68.82%), vasopressor (41.09% vs 58.68%), RRT (6.46% vs 17.79%), antiarrhythmic (17.79% vs 6.49%), and beta-blocker (8.53% vs 3.34%) in the survival group were lower than the death group. The mean age of the survival group was higher than the death group (78.00 years vs 75.77 years). In the samples from MIMIC-IV database, 5094 (84.93%) subjects were survived at the end of the follow-up. The percentages of patients receiving vasopressor (67.26% vs 46.54%), RRT (20.91% vs 8.11%), antiarrhythmic (26.00% vs 17.37%), and beta-blocker (21.90% vs 7.99%) in the survival group were lower than the death group. Participants in the survival group had lower age than the death group (76.73 years vs 74.05 years). The detailed information of participants was presented in Table 1. In the samples from eICU-CRD, 996 participants were survived and 233 were dead. The percentages of people received ventilation in the survival group was lower than the death group (27.51% vs 46.19%). The mean age of the survival group was lower than the death group (73.50 years vs 75.93 years) (Supplementary Table 1).

**Table 1:** The characteristics of patients from MIMIC-III and MIMIC-IV in the survival group and death group

Variables	MIMIC-IV				MIMIC-III			
	Total (*n* = 5998)	Survival(*n* = 5094)	Death (*n* = 904)	*P*	Total (*n* = 4238)	Survival (*n* = 3558)	Death (*n* = 680)	*P*
Ventilation, n (%)				< 0.001				< 0.001
No	533 (8.89)	498 (9.78)	35 (3.87)		1925 (45.42)	1713 (48.15)	212 (31.18)	
Yes	5465 (91.11)	4596 (90.22)	869 (96.13)		2313 (54.58)	1845 (51.85)	468 (68.82)	
Vasopressor, n (%)				< 0.001				< 0.001
No	3019 (50.33)	2723 (53.46)	296 (32.74)		2377 (56.09)	2096 (58.91)	281 (41.32)	
Yes	2979 (49.67)	2371 (46.54)	608 (67.26)		1861 (43.91)	1462 (41.09)	399 (58.68)	
First care unit, n (%)				< 0.001				< 0.001
CCU	1469 (24.49)	1260 (24.73)	209 (23.12)		1081 (25.51)	918 (25.80)	163 (23.97)	
CVICU	1382 (23.04)	1297 (25.46)	85 (9.40)		963 (22.72)	901 (25.32)	62 (9.12)	
MICU	2075 (34.59)	1681 (33.00)	394 (43.58)		1529 (36.08)	1223 (34.37)	306 (45.00)	
Other	563 (9.39)	445 (8.74)	118 (13.05)		225 (5.31)	182 (5.12)	43 (6.32)	
SICU	509 (8.49)	411 (8.07)	98 (10.84)		440 (10.38)	334 (9.39)	106 (15.59)	
Gender, n (%)				0.085				0.918
Female	2610 (43.51)	2193 (43.05)	417 (46.13)		1896 (44.74)	1593 (44.77)	303 (44.56)	
Male	3388 (56.49)	2901 (56.95)	487 (53.87)		2342 (55.26)	1965 (55.23)	377 (55.44)	
Race, n (%)				< 0.001				< 0.001
Black	415 (6.92)	354 (6.95)	61 (6.75)		242 (5.71)	218 (6.13)	24 (3.53)	
Other	509 (8.49)	441 (8.66)	68 (7.52)		241 (5.69)	208 (5.85)	33 (4.85)	
Unknown	651 (10.85)	499 (9.80)	152 (16.81)		464 (10.95)	357 (10.03)	107 (15.74)	
White	4423 (73.74)	3800 (74.60)	623 (68.92)		3291 (77.65)	2775 (77.99)	516 (75.88)	
Insurance, n (%)				0.063				0.006
Medicare	3911 (65.21)	3297 (64.72)	614 (67.92)		3480 (82.11)	2893 (81.31)	587 (86.32)	
Other	2087 (34.79)	1797 (35.28)	290 (32.08)		152 (3.59)	136 (3.82)	16 (2.35)	
Marital status, n (%)				0.417				0.043
Divorced	447 (7.45)	386 (7.58)	61 (6.75)		235 (5.77)	209 (6.07)	26 (4.11)	
Married	2970 (49.52)	2525 (49.57)	445 (49.23)		2078 (50.99)	1742 (50.61)	336 (53.08)	
Single	1150 (19.17)	985 (19.34)	165 (18.25)		631 (15.48)	545 (15.83)	86 (13.59)	
Widowed	1431 (23.86)	1198 (23.52)	233 (25.77)		1060 (26.01)	892 (25.92)	168 (26.54)	
RRT, n (%)				< 0.001				< 0.001
No	5396 (89.96)	4681 (91.89)	715 (79.09)		3887 (91.72)	3328 (93.54)	559 (82.21)	
Yes	602 (10.04)	413 (8.11)	189 (20.91)		351 (8.28)	230 (6.46)	121 (17.79)	
Antiarrhythmic, n (%)				< 0.001				< 0.001
No	4878 (81.33)	4209 (82.63)	669 (74.00)		3887 (91.72)	3328 (93.54)	559 (82.21)	
Yes	1120 (18.67)	885 (17.37)	235 (26.00)		351 (8.28)	230 (6.46)	121 (17.79)	
Antiplatelet, n (%)				1.000				1.000
No	5986 (99.80)	5083 (99.78)	903 (99.89)		4237 (99.98)	3557 (99.97)	680 (100.00)	
Yes	12 (0.20)	11 (0.22)	1 (0.11)		1 (0.02)	1 (0.03)	0 (0.00)	
Anticoagulation, n (%)				0.008				0.131
No	2603 (43.40)	2247 (44.11)	356 (39.38)		3123 (73.69)	2606 (73.24)	517 (76.03)	
Yes	3395 (56.60)	2847 (55.89)	548 (60.62)		1115 (26.31)	952 (26.76)	163 (23.97)	
Beta-blocker, n (%)				< 0.001				< 0.001
No	5393 (89.91)	4687 (92.01)	706 (78.10)		4061 (95.82)	3439 (96.66)	622 (91.47)	
Yes	605 (10.09)	407 (7.99)	198 (21.90)		177 (4.18)	119 (3.34)	58 (8.53)	
CABG, n (%)				< 0.001				< 0.001
No	5331 (88.88)	4455 (87.46)	876 (96.90)		3651 (86.15)	2992 (84.09)	659 (96.91)	
Yes	667 (11.12)	639 (12.54)	28 (3.10)		587 (13.85)	566 (15.91)	21 (3.09)	
Catheter, n (%)				0.024				0.062
No	5936 (98.97)	5035 (98.84)	901 (99.67)		4148 (97.88)	3476 (97.70)	672 (98.82)	
Yes	62 (1.03)	59 (1.16)	3 (0.33)		90 (2.12)	82 (2.30)	8 (1.18)	
Diuretic, n (%)				0.100				0.295
No	2263 (37.73)	1944 (38.16)	319 (35.29)		2865 (67.60)	2417 (67.93)	448 (65.88)	
Yes	3735 (62.27)	3150 (61.84)	585 (64.71)		1373 (32.40)	1141 (32.07)	232 (34.12)	
Age, years, Mean ± SD	74.45 ± 11.50	74.05 ± 11.51	76.73 ± 11.20	< 0.001	76.13 ± 10.74	75.77 ± 10.70	78.00 ± 10.75	< 0.001
Weight, kg, Mean ± SD	83.05 ± 25.08	83.54 ± 25.13	80.25 ± 24.60	< 0.001	81.01 ± 24.12	81.57 ± 24.29	78.18 ± 23.05	0.002
Heart rate, bpm, Mean ± SD	88.82 ± 21.70	88.08 ± 21.34	92.98 ± 23.21	< 0.001	88.20 ± 20.52	87.66 ± 19.98	90.96 ± 22.99	< 0.001
Systolic blood pressure, mmHg, Mean ± SD	119.92 ± 23.91	120.23 ± 23.58	118.20 ± 25.65	0.027	119.60 ± 24.01	119.80 ± 23.47	118.57 ± 26.62	0.262
Diastolic blood pressure, mmHg, Mean ± SD	65.71 ± 18.23	65.64 ± 17.90	66.10 ± 19.96	0.521	60.81 ± 16.52	61.06 ± 16.40	59.50 ± 17.07	0.025
Respiratory rate, Mean ± SD	19.64 ± 6.01	19.39 ± 5.88	21.09 ± 6.54	< 0.001	18.98 ± 6.10	18.61 ± 5.94	20.92 ± 6.57	< 0.001
Temperature, ℃, Mean ± SD	36.58 ± 0.74	36.59 ± 0.71	36.52 ± 0.91	0.038	36.47 ± 0.93	36.49 ± 0.87	36.39 ± 1.19	0.034
SpO_2_, %, Mean ± SD	96.56 ± 4.47	96.70 ± 4.16	95.76 ± 5.85	< 0.001	96.99 ± 4.92	97.13 ± 4.85	96.26 ± 5.25	< 0.001
Charlson comorbidity index, M (Q_1_,Q_3_)	4.00 (2.00, 5.00)	4.00 (2.00, 5.00)	4.00 (3.00, 6.00)	< 0.001	7.01 ± 2.04	6.92 ± 2.00	7.46 ± 2.17	< 0.001
SAPSII, Mean ± SD	41.14 ± 12.57	39.52 ± 11.61	50.26 ± 13.80	< 0.001	41.66 ± 12.60	39.88 ± 11.43	51.00 ± 14.20	< 0.001
GCS, Mean ± SD	13.57 ± 2.78	13.68 ± 2.66	12.98 ± 3.32	< 0.001	13.68 ± 2.68	13.85 ± 2.48	12.83 ± 3.44	< 0.001
WBC, K/uL, M (Q_1_,Q_3_)	10.80 (7.80, 15.00)	10.50 (7.70, 14.60)	12.20 (8.60, 17.50)	< 0.001	11.20 (8.20, 15.10)	11.00 (8.10, 14.80)	12.30 (8.40, 17.80)	< 0.001
Platelet, K/uL, M (Q_1_,Q_3_)	184.00 (135.00, 244.00)	183.00 (135.00, 242.00)	192.00 (133.00, 256.50)	0.035	196.00 (143.00, 261.00)	195.00 (143.00, 260.00)	201.00 (144.00, 265.00)	0.434
Hemoglobin, g/dL, Mean ± SD	10.32 ± 2.19	10.35 ± 2.20	10.18 ± 2.16	0.040	10.48 ± 1.92	10.46 ± 1.92	10.56 ± 1.93	0.211
RDW, %, Mean ± SD	15.68 ± 2.32	15.53 ± 2.23	16.53 ± 2.63	< 0.001	15.52 ± 1.98	15.39 ± 1.91	16.21 ± 2.20	< 0.001
Creatinine, mg/dL, M (Q_1_,Q_3_)	1.20 (0.90, 1.90)	1.20 (0.90, 1.80)	1.50 (1.00, 2.50)	< 0.001	1.20 (0.90, 1.80)	1.10 (0.80, 1.70)	1.50 (1.00, 2.30)	< 0.001
INR, M (Q_1_,Q_3_)	1.50 (1.20, 1.90)	1.40 (1.20, 1.90)	1.50 (1.30, 2.20)	< 0.001	1.50 (1.30, 2.00)	1.50 (1.30, 1.90)	1.60 (1.30, 2.10)	< 0.001
PT, sec, M (Q_1_,Q_3_)	16.00 (13.70, 20.90)	15.90 (13.70, 20.40)	16.90 (13.90, 23.50)	< 0.001	15.90 (14.10, 19.20)	15.80 (14.00, 19.10)	16.25 (14.20, 20.05)	0.011
PTT, sec, M (Q_1_,Q_3_)	33.40 (28.70, 42.20)	33.20 (28.60, 41.60)	34.95 (29.65, 46.10)	< 0.001	34.30 (29.00, 44.50)	34.00 (28.80, 43.80)	35.80 (29.80, 47.20)	< 0.001
BUN, mg/dL, M (Q_1_,Q_3_)	28.00 (19.00, 44.00)	26.00 (18.00, 42.00)	37.00 (24.00, 57.00)	< 0.001	27.00 (18.00, 44.00)	26.00 (18.00, 42.00)	36.00 (24.00, 53.00)	< 0.001
Glucose, mg/dL, M (Q_1_,Q_3_)	132.00 (107.00, 170.00)	131.00 (107.00, 167.00)	141.00 (110.00, 183.00)	< 0.001	131.50 (108.00, 168.00)	130.00 (108.00, 166.00)	139.00 (108.00, 181.00)	0.003
Anion gap, mEq/L, Mean ± SD	15.00 ± 4.22	14.67 ± 3.99	16.85 ± 4.98	< 0.001	14.45 ± 3.79	14.12 ± 3.58	16.14 ± 4.37	< 0.001
Urine output, ml, M (Q_1_,Q_3_)	1400.00 (830.00, 2255.00)	1485.00 (925.00, 2350.00)	928.00 (456.50, 1680.00)	< 0.001	1510.50 (898.00, 2360.00)	1615.00 (989.00, 2453.00)	996.50 (515.00, 1668.00)	< 0.001
Sodium, mEq/L, Mean ± SD	137.47 ± 5.40	137.44 ± 5.29	137.64 ± 5.95	0.336	138.13 ± 4.94	138.07 ± 4.86	138.41 ± 5.39	0.137
COPD, n (%)				0.099				0.120
No	5224 (87.10)	4452 (87.40)	772 (85.40)		4049 (95.54)	3407 (95.76)	642 (94.41)	
Yes	774 (12.90)	642 (12.60)	132 (14.60)		189 (4.46)	151 (4.24)	38 (5.59)	

*SD* Standard deviation, *M* Median, *Q*_*1*_ 1st Quartile, *Q*_*3*_ 3st Quartile, *CCU* Coronary care unit, *CVICU* Cardiac vascular ICU, *MICU* Medical ICU, *SICU* Surgical ICU, *RRT* Renal replacement therapies, *CABG* Coronary artery bypass grafting, *SpO*_*2*_ Oxygen saturation, *SAPSII* Simplified acute physiology score, *GCS* Glasgow coma scale, *WBC* White blood cells, *RDW* Red blood cell distribution width, *INR* International normalized ratio, *PT* Prothrombin time, *PTT* Partial thromboplastin time, *BUN* Blood urea nitrogen, *COPD* Chronic obstructive pulmonary disease

### Construction of the prediction model for in-hospital death in patients with HF and AF

All the samples from MIMIC-IV database were randomly divided into the training set and the testing set at a ratio of 7:3. The baseline data of the participants in the training set and testing set were shown in Table 2. The results of univariate logistical regression model revealed that ventilation, vasopressors, first care unit, race, insurance, RRT, antiarrhythmic, antiplatelet, anticoagulation, beta-blocker, CABG, age, heart rate, systolic blood pressure, respiratory rate, temperature, SpO_2_, Charlson comorbidity index, SAPSII, WBC, platelet, RDW, creatinine, INR, PT, PTT, BUN, glucose, anion gap, urine output and COPD might be predictors for in-hospital death in patients with HF and AF. The final formula of prediction model was shown as follows:.

**Table 2:** The characteristics of patients in the survival group and death group of the training set and testing set

Variables	Training set				Testing set				
	Total (*n* = 4199)	Survival (*n* = 3580)	Death (*n* = 619)	*P*	Total (*n* = 1799)	Survival (*n* = 1514)	Death (*n* = 285)	*P*	*P*
Ventilation, n (%)				< 0.001				< 0.001	0.543
No	368 (8.76)	341 (9.53)	27 (4.36)		165 (9.17)	157 (10.37)	8 (2.81)		
Yes	3831 (91.24)	3239 (90.47)	592 (95.64)		1634 (90.83)	1357 (89.63)	277 (97.19)		
Vasopressor, n (%)				< 0.001				< 0.001	0.757
No	2109 (50.23)	1914 (53.46)	195 (31.50)		910 (50.58)	809 (53.43)	101 (35.44)		
Yes	2090 (49.77)	1666 (46.54)	424 (68.50)		889 (49.42)	705 (46.57)	184 (64.56)		
First care unit, n (%)				< 0.001				< 0.001	0.485
CCU	1017 (24.22)	868 (24.25)	149 (24.07)		452 (25.13)	392 (25.89)	60 (21.05)		
CVICU	980 (23.34)	921 (25.73)	59 (9.53)		402 (22.35)	376 (24.83)	26 (9.12)		
MICU	1450 (34.53)	1187 (33.16)	263 (42.49)		625 (34.74)	494 (32.63)	131 (45.96)		
Other	396 (9.43)	313 (8.74)	83 (13.41)		167 (9.28)	132 (8.72)	35 (12.28)		
SICU	356 (8.48)	291 (8.13)	65 (10.50)		153 (8.50)	120 (7.93)	33 (11.58)		
Gender, n (%)				0.447				0.057	0.070
Female	1800 (42.87)	1526 (42.63)	274 (44.26)		810 (45.03)	667 (44.06)	143 (50.18)		
Male	2399 (57.13)	2054 (57.37)	345 (55.74)		989 (54.97)	847 (55.94)	142 (49.82)		
Race, n (%)				< 0.001				0.021	0.143
Black	280 (6.67)	248 (6.93)	32 (5.17)		135 (7.50)	106 (7.00)	29 (10.18)		
Other	362 (8.62)	313 (8.74)	49 (7.92)		147 (8.17)	128 (8.45)	19 (6.67)		
Unknown	465 (11.07)	353 (9.86)	112 (18.09)		186 (10.34)	146 (9.64)	40 (14.04)		
White	3092 (73.64)	2666 (74.47)	426 (68.82)		1331 (73.99)	1134 (74.90)	197 (69.12)		
Insurance, n (%)				0.025				0.987	0.721
Medicare	2744 (65.35)	2315 (64.66)	429 (69.31)		1167 (64.87)	982 (64.86)	185 (64.91)		
other	1455 (34.65)	1265 (35.34)	190 (30.69)		632 (35.13)	532 (35.14)	100 (35.09)		
Marital status, n (%)				0.666				0.497	0.653
Divorced	318 (7.57)	277 (7.74)	41 (6.62)		129 (7.17)	109 (7.20)	20 (7.02)		
Married	2072 (49.35)	1769 (49.41)	303 (48.95)		898 (49.92)	756 (49.93)	142 (49.82)		
Single	800 (19.05)	683 (19.08)	117 (18.90)		350 (19.46)	302 (19.95)	48 (16.84)		
Widowed	1009 (24.03)	851 (23.77)	158 (25.53)		422 (23.46)	347 (22.92)	75 (26.32)		
RRT, n(%)				< 0.001				< 0.001	0.739
No	3772 (89.83)	3295 (92.04)	477 (77.06)		1624 (90.27)	1386 (91.55)	238 (83.51)		
Yes	427 (10.17)	285 (7.96)	142 (22.94)		175 (9.73)	128 (8.45)	47 (16.49)		
Antiarrhythmic, n (%)				< 0.001				0.003	0.423
No	3407 (81.14)	2953 (82.49)	454 (73.34)		1471 (81.77)	1256 (82.96)	215 (75.44)		
Yes	792 (18.86)	627 (17.51)	165 (26.66)		328 (18.23)	258 (17.04)	70 (24.56)		
Antiplatelet, n (%)				1.000				1.000	1.000
No	4190 (99.79)	3572 (99.78)	618 (99.84)		1796 (99.83)	1511 (99.80)	285 (100.00)		
Yes	9 (0.21)	8 (0.22)	1 (0.16)		3 (0.17)	3 (0.20)	0 (0.00)		
Anticoagulation, n (%)				0.034				0.115	0.081
No	1826 (43.49)	1581 (44.16)	245 (39.58)		777 (43.19)	666 (43.99)	111 (38.95)		
Yes	2373 (56.51)	1999 (55.84)	374 (60.42)		1022 (56.81)	848 (56.01)	174 (61.05)		
Beta-blocker, n (%)				< 0.001				< 0.001	0.812
No	3780 (90.02)	3305 (92.32)	475 (76.74)		1613 (89.66)	1382 (91.28)	231 (81.05)		
Yes	419 (9.98)	275 (7.68)	144 (23.26)		186 (10.34)	132 (8.72)	54 (18.95)		
CABG, n (%)				< 0.001				< 0.001	0.153
No	3725 (88.71)	3123 (87.23)	602 (97.25)		1606 (89.27)	1332 (87.98)	274 (96.14)		
Yes	474 (11.29)	457 (12.77)	17 (2.75)		193 (10.73)	182 (12.02)	11 (3.86)		
Catheter, n (%)				0.038				0.711	0.657
No	4151 (98.86)	3534 (98.72)	617 (99.68)		1785 (99.22)	1501 (99.14)	284 (99.65)		
Yes	48 (1.14)	46 (1.28)	2 (0.32)		14 (0.78)	13 (0.86)	1 (0.35)		
Diuretic, n (%)				0.210				0.281	0.239
No	1587 (37.79)	1367 (38.18)	220 (35.54)		676 (37.58)	577 (38.11)	99 (34.74)		
Yes	2612 (62.21)	2213 (61.82)	399 (64.46)		1123 (62.42)	937 (61.89)	186 (65.26)		
Age, years, Mean ± SD	74.43 ± 11.46	74.03 ± 11.50	76.76 ± 10.98	< 0.001	74.50 ± 11.60	74.10 ± 11.54	76.67 ± 11.68	< 0.001	0.091
Weight, kg, Mean ± SD	82.88 ± 24.77	83.26 ± 24.75	80.66 ± 24.81	0.016	83.43 ± 25.78	84.20 ± 26.01	79.35 ± 24.16	0.004	0.717
Heart rate, Mean ± SD	88.82 ± 21.78	88.15 ± 21.48	92.67 ± 23.06	< 0.001	88.81 ± 21.52	87.90 ± 20.99	93.65 ± 23.57	< 0.001	0.195
Systolic, mmHg, Mean ± SD	119.94 ± 23.96	120.35 ± 23.71	117.60 ± 25.26	0.012	119.87 ± 23.80	119.94 ± 23.27	119.51 ± 26.46	0.799	0.333
Diastolic, mmHg, Mean ± SD	65.80 ± 18.25	65.71 ± 17.85	66.35 ± 20.46	0.465	65.50 ± 18.17	65.49 ± 18.04	65.56 ± 18.88	0.953	0.360
Respiratory rate, Mean ± SD	19.60 ± 5.95	19.31 ± 5.81	21.28 ± 6.46	< 0.001	19.74 ± 6.15	19.56 ± 6.02	20.68 ± 6.72	0.009	0.579
Temperature, ℃, Mean ± SD	36.57 ± 0.75	36.58 ± 0.72	36.50 ± 0.94	0.038	36.60 ± 0.71	36.61 ± 0.69	36.57 ± 0.83	0.536	0.138
SpO_2_, %, Mean ± SD	96.57 ± 4.38	96.76 ± 3.96	95.51 ± 6.18	< 0.001	96.54 ± 4.68	96.58 ± 4.61	96.31 ± 5.02	0.371	0.547
Charlson comorbidity index, M (Q_1_,Q_3_)	4.00 (2.00, 5.00)	4.00 (2.00, 5.00)	4.00 (3.00, 6.00)	< 0.001	4.00 (2.00, 5.00)	4.00 (2.00, 5.00)	4.00 (3.00, 6.00)	< 0.001	0.619
SAPSII, Mean ± SD	41.20 ± 12.53	39.57 ± 11.53	50.62 ± 13.89	< 0.001	40.99 ± 12.65	39.40 ± 11.80	49.46 ± 13.60	< 0.001	0.161
GCS, Mean ± SD	13.56 ± 2.80	13.66 ± 2.67	12.99 ± 3.36	< 0.001	13.60 ± 2.74	13.72 ± 2.62	12.95 ± 3.24	< 0.001	0.217
WBC, K/uL, M (Q_1_,Q_3_)	10.80 (7.90, 15.00)	10.60 (7.75, 14.60)	12.50 (8.90, 18.00)	< 0.001	10.60 (7.50, 14.80)	10.40 (7.50, 14.60)	11.20 (7.70, 16.60)	0.045	0.607
Platelet, K/uL, M (Q_1_,Q_3_)	183.00 (135.00, 241.00)	182.00 (135.00, 239.00)	192.00 (139.00, 254.00)	0.007	185.00 (135.00, 250.00)	184.00 (137.00, 249.00)	190.00 (126.00, 263.00)	0.821	0.716
Hemoglobin, g/dL, Mean ± SD	10.34 ± 2.22	10.35 ± 2.22	10.30 ± 2.22	0.576	10.27 ± 2.13	10.33 ± 2.15	9.94 ± 2.01	0.004	0.614
RDW, %, Mean ± SD	15.65 ± 2.31	15.51 ± 2.24	16.45 ± 2.56	< 0.001	15.74 ± 2.34	15.56 ± 2.20	16.69 ± 2.79	< 0.001	0.110
Creatinine blood, mg/dL, M (Q_1_,Q_3_)	1.20 (0.90, 1.90)	1.20 (0.90, 1.80)	1.60 (1.10, 2.50)	< 0.001	1.20 (0.90, 1.90)	1.20 (0.90, 1.80)	1.40 (1.00, 2.30)	< 0.001	0.230
INR, M (Q_1_,Q_3_)	1.50 (1.20, 1.90)	1.40 (1.20, 1.90)	1.60 (1.30, 2.30)	< 0.001	1.50 (1.20, 1.90)	1.50 (1.20, 1.90)	1.50 (1.20, 2.00)	0.419	0.058
PT, sec, M (Q_1_,Q_3_)	15.90 (13.70, 21.00)	15.80 (13.70, 20.20)	17.10 (13.90, 24.40)	< 0.001	16.10 (13.70, 20.70)	16.00 (13.70, 20.60)	16.30 (13.80, 21.20)	0.408	0.054
PTT, sec, M (Q_1_,Q_3_)	33.40 (28.70, 42.50)	33.10 (28.60, 41.60)	36.00 (29.90, 48.70)	< 0.001	33.50 (28.70, 41.40)	33.50 (28.70, 41.20)	33.50 (29.10, 42.10)	0.549	0.465
BUN, mg/dL, M (Q_1_,Q_3_)	28.00 (19.00, 44.00)	26.00 (18.00, 42.00)	38.00 (24.00, 58.00)	< 0.001	28.00 (18.00, 45.00)	27.00 (18.00, 43.00)	34.00 (24.00, 56.00)	< 0.001	0.985
Glucose, mg/dL, M (Q_1_,Q_3_)	132.00 (108.00, 169.00)	131.00 (107.00, 166.00)	143.00 (112.00, 183.00)	< 0.001	132.00 (106.00, 171.00)	131.00 (106.00, 169.00)	137.00 (107.00, 184.00)	0.058	0.117
Anion gap, mEq/L, Mean ± SD	15.00 ± 4.18	14.67 ± 3.96	16.96 ± 4.82	< 0.001	15.00 ± 4.32	14.69 ± 4.04	16.62 ± 5.31	< 0.001	0.748
Urine output, ml, M (Q_1_,Q_3_)	1395.00 (825.00, 2240.00)	1465.00 (909.00, 2315.00)	910.00 (405.00, 1669.00)	< 0.001	1420.00 (852.00, 2355.00)	1512.50 (942.00, 2425.00)	985.00 (565.00, 1720.00)	< 0.001	0.976
Sodium, mEq/L, Mean ± SD	137.49 ± 5.48	137.48 ± 5.43	137.52 ± 5.76	0.864	137.42 ± 5.20	137.33 ± 4.95	137.90 ± 6.35	0.155	0.991
COPD, n (%)				0.508				0.048	0.234
No	3657 (87.09)	3123 (87.23)	534 (86.27)		1567 (87.10)	1329 (87.78)	238 (83.51)		
Yes	542 (12.91)	457 (12.77)	85 (13.73)		232 (12.90)	185 (12.22)	47 (16.49)		

*SD* Standard deviation, *M* Median, *Q1* 1st Quartile, *Q3* 3st Quartile, *CCU* Coronary care unit, *CVICU* Cardiac vascular ICU, *MICU* Medical ICU, *SICU* Surgical ICU, *RRT* Renal replacement therapies, *CABG* Coronary artery bypass grafting, *SpO2* Oxygen saturation, *SAPSII* Simplified acute physiology score, *GCS* Glasgow coma scale, *WBC* White blood cells, *RDW* Red blood cell distribution width, *INR* International normalized ratio, *PT* Prothrombin time, *PTT* Partial thromboplastin time, *BUN* Blood urea nitrogen, *COPD* Chronic obstructive pulmonary disease

$$\begin{array}{c}1\mathrm n\left(\frac{\mathrm p}{1-\mathrm p}\right)=-5.5310+0.0474\times\mathrm{SAPSII}+0.1121\times\mathrm{RDW}-0.4192\times\mathrm{Beta}-\mathrm{blocker}\;\left(\mathrm{no}\right)-0.4175\times\mathrm{Race}\;(\mathrm{Black})\\-0.1878\times\mathrm{Race}\;\left(\mathrm{others}\right)+0.6578\times\mathrm{Race}\;\left(\mathrm{unknown}\right)+0.0308\times\mathrm{Respiratoryrate}\\\begin{array}{c}-0.00022\times\mathrm{Urineoutput}+0.7242\times\mathrm{CABG}\;\left(\mathrm{no}\right)+0.0967\times\mathrm{Charlsoncomorbidityindex}\\\begin{array}{c}-0.0239\times\mathrm{Spo}2+0.00333\times\mathrm{PPT}-0.1961\times\mathrm{Antiarrhytmic}\;\left(\mathrm{no}\right)+0.0133\times\mathrm{Age}\\\begin{array}{c}-0.3853\times\mathrm{RRT}\;\left(\mathrm{no}\right)-0.2248\times\mathrm{Creatinine}+0.0538\times\mathrm{Anion gap}\\-0.1747\times\mathrm{Anticoagulation}\end{array}\end{array}\end{array}\end{array}$$

**Fig. 2:**
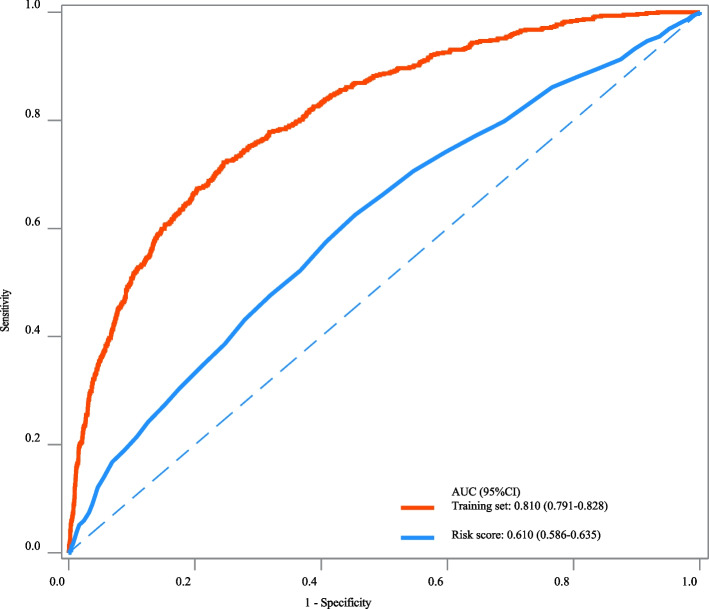
The ROC curve of our prediction model and the previous risk score in the training set

**Fig. 3:**
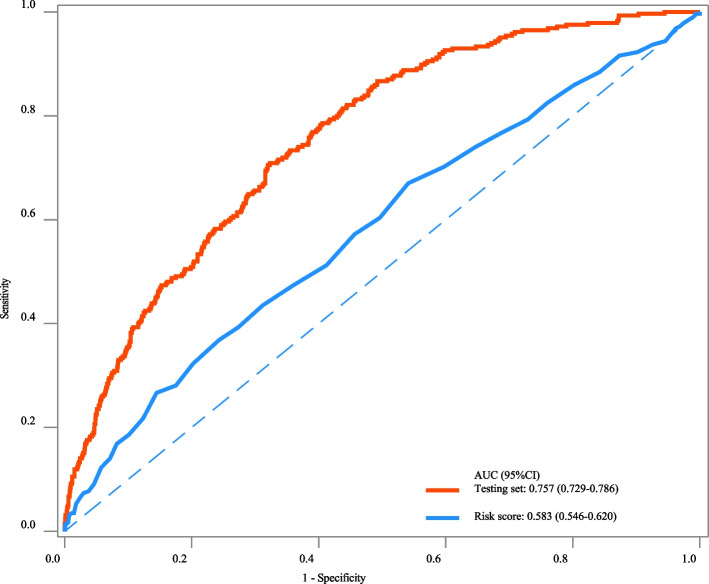
The ROC curve of our prediction model and the previous risk score in the testing set

**Fig. 4:**
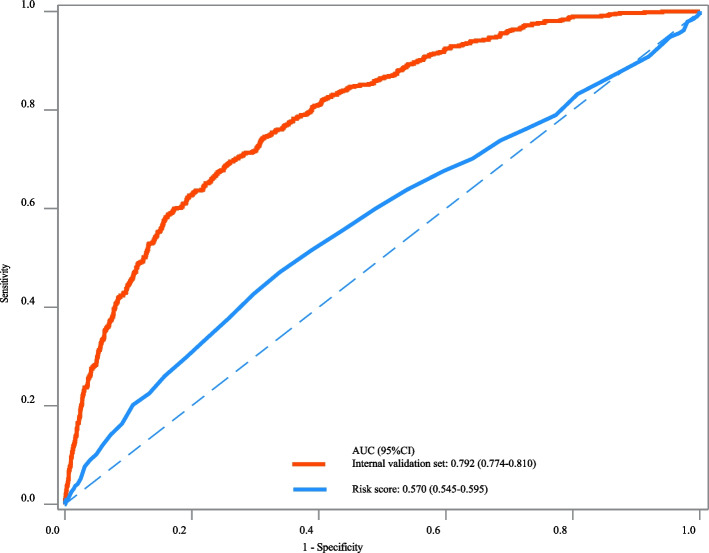
The ROC curve of our prediction model and the previous risk score in the internal validation set

**Fig. 5:**
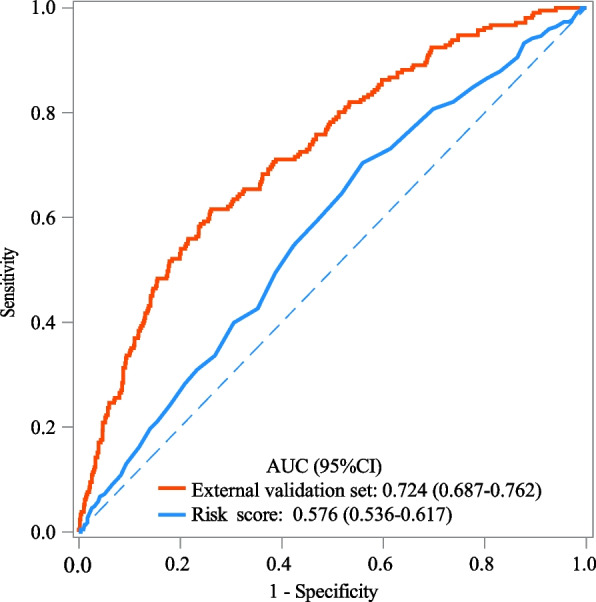
The ROC curve of our prediction model and the previous risk score in the external validation set

**Fig. 6:**
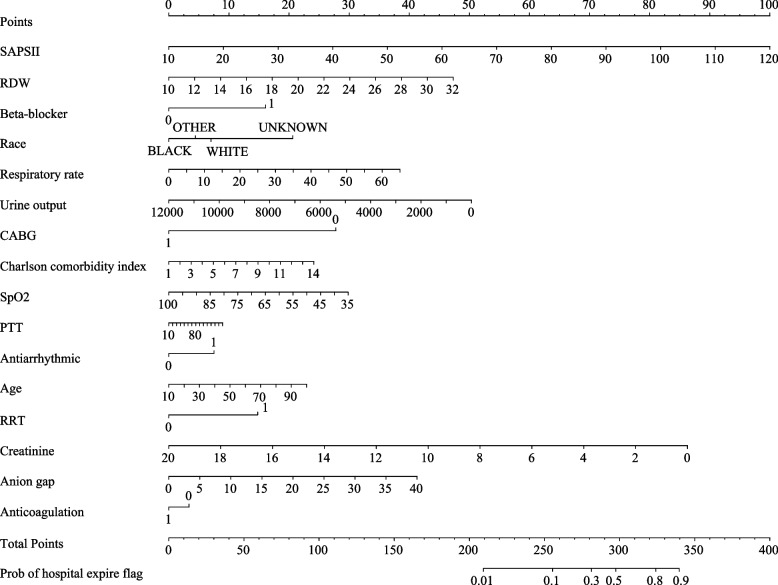
The nomogram of our prediction model

The AUC, specificity, NPV and accuracy of our prediction model in the training set were 0.810 (95%CI: 0.791–0.828), 0.755 (95%CI: 0.740–0.769), 0.940 (95%CI: 0.931–0.948), and 0.750 (95%CI: 0.736–0.763). The AUC, specificity, and NPV of our prediction model in the testing set were 0.757 (95%CI: 0.729–0.786), 0.760 (95%CI: 0.738–0.782), and 0.906 (95%CI: 0.889–0.922). The AUC of our prediction model in the internal validation set was 0.792 (95%CI: 0.774–0.810) and 0.724 (95%CI: 0.687–0.762) in the external validation set (Table 3). The ROC curves of our prediction model and the previous risk score in the training set, testing set, internal validation set, and external validation set were exhibited in Figs. 2, 3, 4 and 5, respectively. The calibration curves of the model in the training set (Supplementary Fig. 1), testing set (Supplementary Fig. 2), internal validation set (Supplementary Fig. 3), and external validation set (Supplementary Fig. 4) revealed that the predictive probabilities of our model for the in-hospital death in patients with HF and AF deviated slightly from the ideal model. The DCA curves revealed that the use of our prediction model increased the net benefit than use no model, suggesting that the model might help the clinicians quickly identify those at high risk of in-hospital mortality (Supplementary Figs. 5, 6, 7 and 8). The nomogram of the prediction model was plotted (Fig. 6). Delong test depicted that the AUCs of our model in the training set, testing set, and internal validation set were higher than the previous risk score (Table 4).

**Table 3:** The predictive values of the models

Dataset	Model/Score	Cut-off	AUC (95%CI)	Sensitivity (95%CI)	Specificity (95%CI)	NPV (95%CI)	PPV (95%CI)	Accuracy (95%CI)
Training set	Our model	0.152	0.810 (0.791–0.828)	0.721 (0.683–0.756)	0.755 (0.740–0.769)	0.940 (0.931–0.948)	0.337 (0.311–0.363)	0.750 (0.736–0.763)
	Risk score	0.0359	0.610 (0.586–0.635)	0.575 (0.535–0.614)	0.592 (0.576–0.608)	0.890 (0.876–0.902)	0.196 (0.178–0.215)	0.590 (0.575–0.605)
Testing set	Our model	0.152	0.757 (0.729–0.786)	0.582 (0.523–0.640)	0.760 (0.738–0.782)	0.906 (0.889–0.922)	0.314 (0.274–0.355)	0.732 (0.711–0.752)
	Risk score	0.0359	0.583 (0.546–0.620)	0.604 (0.544–0.661)	0.504 (0.478–0.529)	0.871 (0.847–0.892)	0.186 (0.162–0.213)	0.520 (0.496–0.543)
Internal validation set	Our model	0.152	0.792 (0.774–0.810)	0.747 (0.713–0.779)	0.679 (0.664–0.695)	0.934 (0.923–0.943)	0.308 (0.286–0.331)	0.690 (0.676–0.704)
	Risk score	0.0359	0.570 (0.545–0.595)	0.557 (0.519–0.595)	0.561 (0.544–0.577)	0.869 (0.854–0.882)	0.195 (0.178–0.213)	0.560 (0.545–0.575)
External validation set	Our model	0.152	0.724 (0.687–0.762)	0.588 (0.518–0.655)	0.760 (0.732–0.787)	0.893 (0.869–0.913)	0.352 (0.302–0.405)	0.729 (0.702–0.754)
	Risk score	0.0359	0.576 (0.536–0.617)	0.646 (0.579–0.708)	0.482 (0.450–0.513)	0.859 (0.827–0.886)	0.218 (0.187–0.252)	0.512 (0.483–0.540)

*AUC* Area under the curve, *CI* Confidence interval, *NPV* Negative predictive value, *PPV* Positive predictive value

**Table 4:** The results of Delong test comparing the predictive value of our model with GWTG-HF risk score

Dataset	AUC Our model	AUC Risk score	Chi-square	*P*
Training set	0.8098	0.6012	226.8630	< 0.0001
Testing set	0.7572	0.5829	82.4924	< 0.0001
Internal validation set	0.7921	0.5701	278.1109	< 0.0001
External validation set	0.7242	0.5735	32.1472	< 0.0001

*GWTG-HF* American Heart Association Get With the Guidelines-Heart Failure, *AUC* Area under the curve, *CI* Confidence interval

## Discussion

In the current study, a prediction model for in-hospital mortality of HF patients with AF was established based on the predictors including race, RRT, antiarrhythmic, anticoagulation, beta-blocker, CABG, age, respiratory rate, SpO_2_, Charlson comorbidity index, SAPSII, RDW, creatinine, PTT, anion gap, and urine output. The prediction model showed good predictive performance with AUC of 0.810 in the training set, 0.757 in the testing set, 0.792 in the internal validation set and 0.724 in the external validation set. The prediction model might provide a useful tool to early identify patients complicated with HF and AF who were at high risk of in-hospital death, and offer timely interventions to improve their prognosis.

At present, several models were established for predicting the mortality of HF patients. Li et al. established a prediction model for in-hospital mortality in ICU patients with HF using machine learning methods, showing good predictive performance [19]. Another multivariable prediction model for the mortality of patients with HF had a C-index of 0.70 [20]. The GWTG-HF risk score is constructed to predict the risk of in-hospital mortality for patients hospitalized with HF based on information concerning patient age, SBP, BUN, HR, serum sodium, COPD and non-African American ethnicity [21]. The GWTG-HF risk score is widely applied to be a prognostic tool for evaluating the mortality of HF patients [22, 23]. The GWTG-HF risk score for participants in this study was also calculated, and the discrimination performance were validated in the samples of our study. The predictive performance of the model in the current study was superior to the GWTG-HF score. Several other risk scores including the Intermountain Risk Score (IMRS) and the Naples score (NS) were established for other heart diseases such as cardiogenic shock or myocardial infarction [24, 25]. Compared with previous models and risk score, our model could quickly identify patients with both HF and AF who were at high risk of in-hospital mortality. We also verify the predictive performance of the model in the internal validation set using the data from MIMIC-III database and external validation set using the data from eICU-CRD. The model had good discrimination ability for HF and AF patients with high risk of in-hospital death. This model combined fast and routinely available variables including demographic characteristic and laboratory characteristics, which seemed to be a promising tool for early and accurate risk stratification in the ICUs. The nomogram was also plotted, and the probability of in-hospital mortality of patients with HF and AF could quickly be obtained. For clinicians, special interventions and care should be applied to those with high risk of mortality in patients with HF and AF.

A previous nomogram revealed that age, AG ≥ 20 mEq/L, RDW ≥ 15.5%, and beta‐blocker were important predictors for the in‐hospital mortality of patients with congestive HF and chronic kidney disease [26]. Wussler et al. conducted a review exploring recent advances and remaining uncertainties regarding risk stratification in acute HF, which identified that age, respiratory rate, oxygen saturation, and creatinine were the most commonly used predictor variables in the described risk scores [27]. There was evidence indicated that antiarrhythmic [28, 29], beta-blockers [30, 31], and anticoagulation [32] were correlated with the prognosis of patients with HF or patients with HF and AF. Charlson comorbidity index, the most extensively studied comorbidity index, was found to be associated with the clinical outcome in patients with HF [33] and an important predictor for 30-day readmission in patients with HF exacerbation and AF [34]. Age and creatinine were also demonstrated to have predictive value for in-hospital mortality in patients with cardiogenic shock [25]. These findings supported the results in the present study, which showed that race, RRT, antiarrhythmic, anticoagulation, beta-blocker, age, respiratory rate, SpO_2_, Charlson comorbidity index, RDW, creatinine, anion gap, and urine output were essential predictors for in-hospital death in patients with HF and AF.

Several limitations were found in this study. Firstly, due to the limitation of the MIMIC database, the data on left ventricular ejection fraction, and lactate level were not reported, which might affect the results. Secondly, electrocardiograms scores have been applied for the prediction of diastolic dysfunction and other diseases in previous studies [35, 36], but the data on electrocardiograms could not been obtained from MIMIC database. Thirdly, patients with missing death information were excluded, which might impact the generalizability of the model. In the future, more studies are needed to verify the findings of our study.

## Conclusions

The present study established a prediction model for in-hospital death mortality of patients with HF complicated with AF. The prediction model had good discriminative ability, and might provide a tool to quickly identify patients with HF complicated with AF who were at high risk of in-hospital mortality.

### Supplementary Information

**Additional file 1: Supplementary Table 1.** The characteristics of patients from eICU in the survival group and death group.

**Additional file 2: Supplementary Figure 1.** The calibration curve of our prediction model in the training set.

**Additional file 3: Supplementary Figure 2. **The calibration curve of our prediction model in the testing set.

**Additional file 4: Supplementary Figure 3.** The calibration curve of our prediction model in the internal validation set.

**Additional file 5: Supplementary Figure 4.** The calibration curve of our prediction model in the external validation set.

**Additional file 6: Supplementary Figure 5.** The DCA curve of our prediction model in the training set.

**Additional file 7: Supplementary Figure 6.** The DCA curve of our prediction model in the testing set.

**Additional file 8: Supplementary Figure 7.** The DCA curve of our prediction model in the internal validation set.

**Additional file 9: Supplementary Figure 8.** The DCA curve of our prediction model in the external validation set.

## Data Availability

The datasets used and/or analyzed during the current study are available from the MIMIC-III and MIMIC-IV database, https://www.physionet.org/content/mimiciii/1.4/, https://www.physionet.org/content/mimiciv/2.2/.

## Abbreviations

AF: Atrial fibrillation
HF: Heart failure
GWTG-HF: Get With the Guidelines-Heart Failure
MIMIC: Medical Information Mart for Intensive Care
ICU: Intensive care unit
CCU: Coronary care unit
CVICU: Cardiac vascular ICU
MICU: Medical ICU
SICU: Surgical ICU
RRT: Renal replacement therapies
CABG: Coronary artery bypass grafting
SpO_2_: Oxygen saturation
SAPS: Simplified acute physiology score
GCS: Glasgow coma scale
WBC: White blood cells
INR: International normalized ratio
PT: Prothrombin time
PTT: Partial thromboplastin time
BUN: Blood urea nitrogen
COPD: Chronic obstructive pulmonary disease
SD: Standard deviation
