# Supplementary material for: Development and validation of a prediction model for in-hospital death in patients with heart failure and atrial fibrillation
Source: BMC Cardiovasc Disord. 2023 Oct 11;23:505. doi: 10.1186/s12872-023-03521-3 (PMC10566083; doi:10.1186/s12872-023-03521-3)
Supplement: Supplementary file 1 — Additional file 1: Supplementary Table 1. The characteristics of patients from eICU in the survival group and death group. [file 12872_2023_3521_MOESM1_ESM.docx]

Supplementary Table 1 The characteristics of patients from eICU in the survival group and death group

|  | | eICU | |  | |
| --- | --- | --- | --- | --- | --- |
| Variables | Total (n=1219) | Survival (n=996) | Death (n=223) | Statistics | *P* |
| Ventilation, n (%) |  |  |  | χ^2^=29.757 | <0.001 |
| No | 842 (69.07) | 722 (72.49) | 120 (53.81) |  |  |
| Yes | 377 (30.93) | 274 (27.51) | 103 (46.19) |  |  |
| Vasopressor, n (%) |  |  |  | χ^2^=35.594 | <0.001 |
| No | 908 (74.49) | 777 (78.01) | 131 (58.74) |  |  |
| Yes | 311 (25.51) | 219 (21.99) | 92 (41.26) |  |  |
| First care unit, n (%) |  |  |  | χ^2^=0.024 | 0.988 |
| MICU | 735 (60.30) | 601 (60.34) | 134 (60.09) |  |  |
| SICU | 73 (5.99) | 60 (6.02) | 13 (5.83) |  |  |
| Other | 411 (33.72) | 335 (33.63) | 76 (34.08) |  |  |
| Gender, n (%) |  |  |  | χ^2^=0.220 | 0.639 |
| Female | 531 (43.56) | 437 (43.88) | 94 (42.15) |  |  |
| Male | 688 (56.44) | 559 (56.12) | 129 (57.85) |  |  |
| Race, n (%) |  |  |  | χ^2^=1.666 | 0.645 |
| Black | 76 (6.23) | 66 (6.63) | 10 (4.48) |  |  |
| Other | 102 (8.37) | 83 (8.33) | 19 (8.52) |  |  |
| Unknown | 57 (4.68) | 45 (4.52) | 12 (5.38) |  |  |
| White | 984 (80.72) | 802 (80.52) | 182 (81.61) |  |  |
| RRT, n (%) |  |  |  | χ^2^=0.307 | 0.579 |
| No | 1126 (92.37) | 922 (92.57) | 204 (91.48) |  |  |
| Yes | 93 (7.63) | 74 (7.43) | 19 (8.52) |  |  |
| Antiarrhythmic, n (%) |  |  |  | χ^2^=0.019 | 0.889 |
| No | 672 (55.13) | 550 (55.22) | 122 (54.71) |  |  |
| Yes | 547 (44.87) | 446 (44.78) | 101 (45.29) |  |  |
| Antiplatelet, n (%) |  |  |  | χ^2^=2.090 | 0.148 |
| No | 974 (79.90) | 788 (79.12) | 186 (83.41) |  |  |
| Yes | 245 (20.10) | 208 (20.88) | 37 (16.59) |  |  |
| Anticoagulation, n (%) |  |  |  | χ^2^=1.011 | 0.315 |
| No | 874 (71.70) | 708 (71.08) | 166 (74.44) |  |  |
| Yes | 345 (28.30) | 288 (28.92) | 57 (25.56) |  |  |
| Beta-blocker, n (%) |  |  |  | χ^2^=1.599 | 0.206 |
| No | 820 (67.27) | 678 (68.07) | 142 (63.68) |  |  |
| Yes | 399 (32.73) | 318 (31.93) | 81 (36.32) |  |  |
| CABG, n (%) |  |  |  | χ^2^=3.815 | 0.051 |
| No | 1184 (97.13) | 963 (96.69) | 221 (99.10) |  |  |
| Yes | 35 (2.87) | 33 (3.31) | 2 (0.90) |  |  |
| Catheter, n (%) |  |  |  | - | 0.599 |
| No | 1213 (99.51) | 990 (99.40) | 223 (100.00) |  |  |
| Yes | 6 (0.49) | 6 (0.60) | 0 (0.00) |  |  |
| Diuretic, n (%) |  |  |  | χ^2^=2.563 | 0.109 |
| No | 757 (62.10) | 629 (63.15) | 128 (57.40) |  |  |
| Yes | 462 (37.90) | 367 (36.85) | 95 (42.60) |  |  |
| Age, years, Mean ± SD | 73.95 ± 11.94 | 73.50 ± 12.19 | 75.93 ± 10.61 | t=-3.00 | 0.003 |
| Weight, kg, Mean ± SD | 88.27 ± 29.35 | 88.90 ± 29.60 | 85.45 ± 28.11 | t=1.59 | 0.113 |
| Heart rate, bpm, Mean ± SD | 96.80 ± 25.93 | 97.09 ± 25.76 | 95.51 ± 26.71 | t=0.82 | 0.412 |
| Systolic blood pressure, mmHg, Mean ± SD | 121.35 ± 28.40 | 122.29 ± 28.38 | 117.17 ± 28.18 | t=2.43 | 0.015 |
| Diastolic blood pressure, mmHg, Mean ± SD | 70.31 ± 20.21 | 70.75 ± 20.26 | 68.36 ± 19.92 | t=1.60 | 0.110 |
| Respiratory rate, Mean ± SD | 21.92 ± 6.65 | 21.90 ± 6.64 | 22.02 ± 6.75 | t=-0.24 | 0.810 |
| Temperature, ℃, Mean ± SD | 36.69 ± 0.90 | 36.74 ± 0.81 | 36.49 ± 1.19 | t=2.93 | 0.004 |
| SpO_2_, %, Mean ± SD | 95.20 ± 6.66 | 95.27 ± 6.55 | 94.87 ± 7.14 | t=0.82 | 0.413 |
| Charlson comorbidity index, M(Q_1_,Q_3_) | 5.83 ± 1.70 | 5.72 ± 1.70 | 6.30 ± 1.67 | t=-4.63 | <0.001 |
| SAPSII, Mean ± SD | 29.00 (22.00, 40.00) | 27.00 (21.00, 36.00) | 41.00 (30.00, 55.00) | Z=11.802 | <0.001 |
| GCS, Mean ± SD | 13.00 (8.00, 15.00) | 14.00 (9.00, 15.00) | 8.00 (3.00, 13.00) | Z=-10.170 | <0.001 |
| WBC, K/uL, M(Q_1_,Q_3_) | 10.60 (8.00, 14.60) | 10.30 (7.90, 14.34) | 12.10 (8.60, 16.50) | Z=3.039 | 0.002 |
| Platelet, K/uL, M(Q_1_,Q_3_) | 194.00 (148.00, 256.00) | 199.00 (151.00, 259.00) | 184.00 (135.00, 239.00) | Z=-2.548 | 0.011 |
| Hemoglobin,g/dL, Mean ± SD | 11.55 ± 2.49 | 11.60 ± 2.47 | 11.35 ± 2.58 | t=1.36 | 0.175 |
| RDW, %, Mean ± SD | 16.05 ± 2.34 | 15.95 ± 2.30 | 16.46 ± 2.49 | t=-2.93 | 0.003 |
| Creatinine, mg/dL, M(Q_1_,Q_3_) | 1.42 (1.00, 2.16) | 1.40 (1.00, 2.10) | 1.71 (1.14, 2.53) | Z=3.464 | <0.001 |
| INR, M(Q_1_,Q_3_) | 1.40 (1.10, 2.00) | 1.30 (1.10, 1.90) | 1.50 (1.20, 2.36) | Z=3.360 | <0.001 |
| PT, sec, M(Q_1_,Q_3_) | 15.90 (13.40, 22.20) | 15.70 (13.20, 21.35) | 17.10 (14.20, 25.40) | Z=3.495 | <0.001 |
| PTT, sec, M(Q_1_,Q_3_) | 33.50 (29.00, 41.80) | 33.20 (28.95, 41.55) | 34.10 (29.60, 42.90) | Z=1.489 | 0.136 |
| BUN, mg/dL, M(Q_1_,Q_3_) | 30.00 (20.00, 47.00) | 29.00 (20.00, 45.00) | 35.00 (24.00, 58.00) | Z=4.497 | <0.001 |
| Glucose, mg/dL, M(Q_1_,Q_3_) | 137.00 (109.00, 186.00) | 135.00 (109.00, 184.00) | 146.00 (115.00, 195.00) | Z=1.614 | 0.107 |
| Anion gap, mEq/L, Mean ± SD | 11.00 (8.00, 14.90) | 11.00 (8.00, 14.50) | 11.00 (9.00, 15.00) | Z=1.823 | 0.068 |
| Urine output, ml, M(Q_1_,Q_3_) | 1329.00 (650.00, 2300.00) | 1439.00 (708.50, 2432.00) | 1030.00 (410.00, 1888.00) | Z=-4.920 | <0.001 |
| Sodium, mEq/L, Mean ± SD | 136.73 ± 5.57 | 136.60 ± 5.50 | 137.33 ± 5.83 | t=-1.78 | 0.076 |
| COPD, n (%) |  |  |  | χ^2^=0.333 | 0.564 |
| No | 947 (77.69) | 777 (78.01) | 170 (76.23) |  |  |
| Yes | 272 (22.31) | 219 (21.99) | 53 (23.77) |  |  |

SD: standard deviation, M: Median, Q1: 1st Quartile, Q3:3st Quartile, CCU: Coronary care unit, CVICU: Cardiac vascular ICU, MICU: medical ICU, SICU: surgical ICU, RRT: Renal replacement therapies, CABG: Coronary artery bypass grafting, SpO2: oxygen saturation, SAPSII: Simplified acute physiology score, GCS: Glasgow coma scale, WBC: White blood cells, RDW: Red blood cell distribution width, INR: International normalized ratio, PT: Prothrombin time, PTT: Partial thromboplastin time, BUN: Blood urea nitrogen, COPD: Chronic obstructive pulmonary disease
