# Supplementary material for: Development and validation of a prediction model for in-hospital death in patients with heart failure and atrial fibrillation
Source: BMC Cardiovasc Disord. 2023 Oct 11;23:505. doi: 10.1186/s12872-023-03521-3 (PMC10566083; doi:10.1186/s12872-023-03521-3)

Supplementary Figure 2 The calibration curve of our prediction model in the testing set.

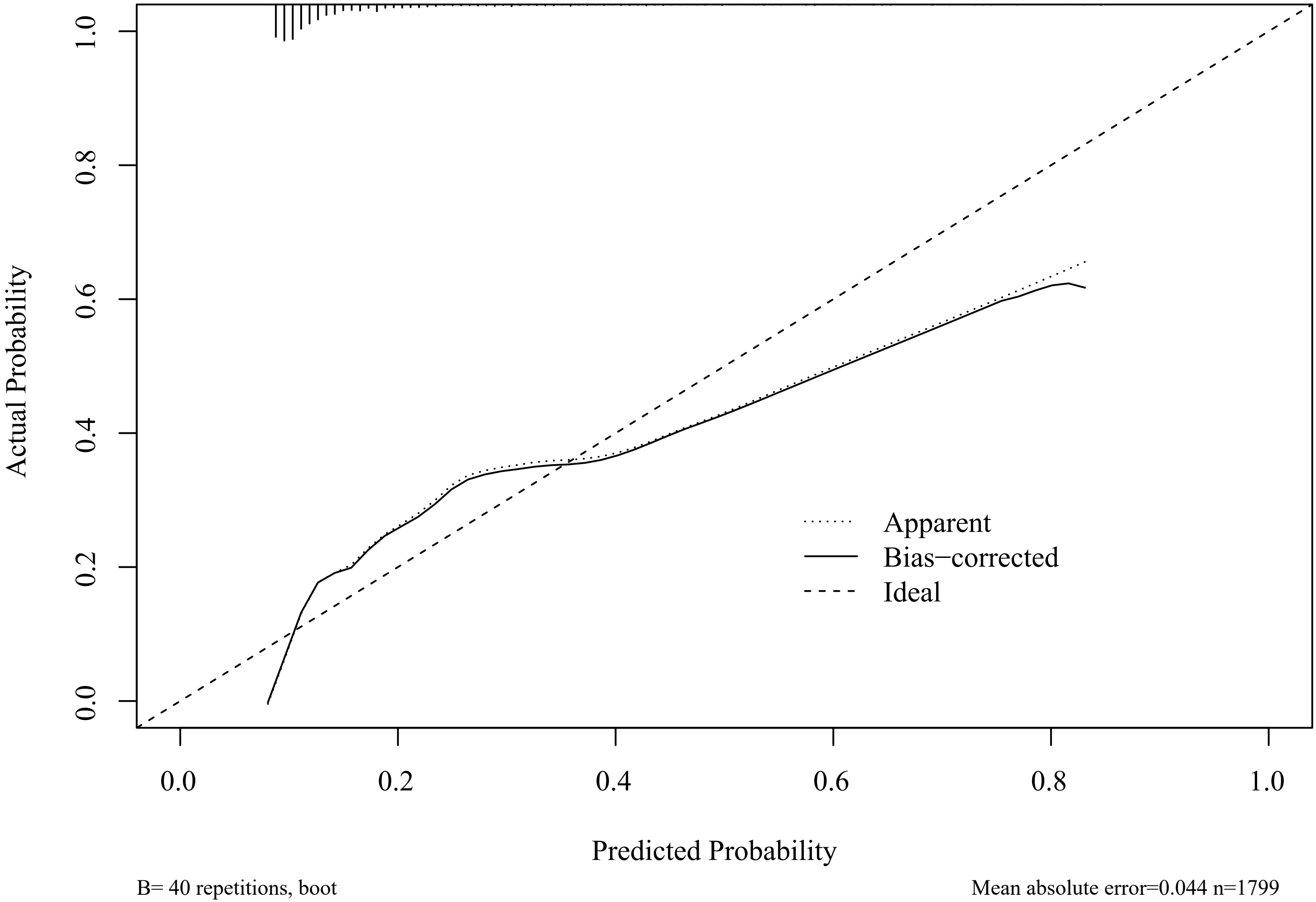

Supplement: Supplementary file 3 — Additional file 3: Supplementary Figure 2. The calibration curve of our prediction model in the testing set. [file 12872_2023_3521_MOESM3_ESM.pdf]
