# Supplementary figures and images for: Development and validation of a prediction model for in-hospital death in patients with heart failure and atrial fibrillation
Source: BMC Cardiovasc Disord. 2023 Oct 11;23:505. doi: 10.1186/s12872-023-03521-3 (PMC10566083; doi:10.1186/s12872-023-03521-3)

Supplementary Figure 5 The DCA curve of our prediction model in the training set.

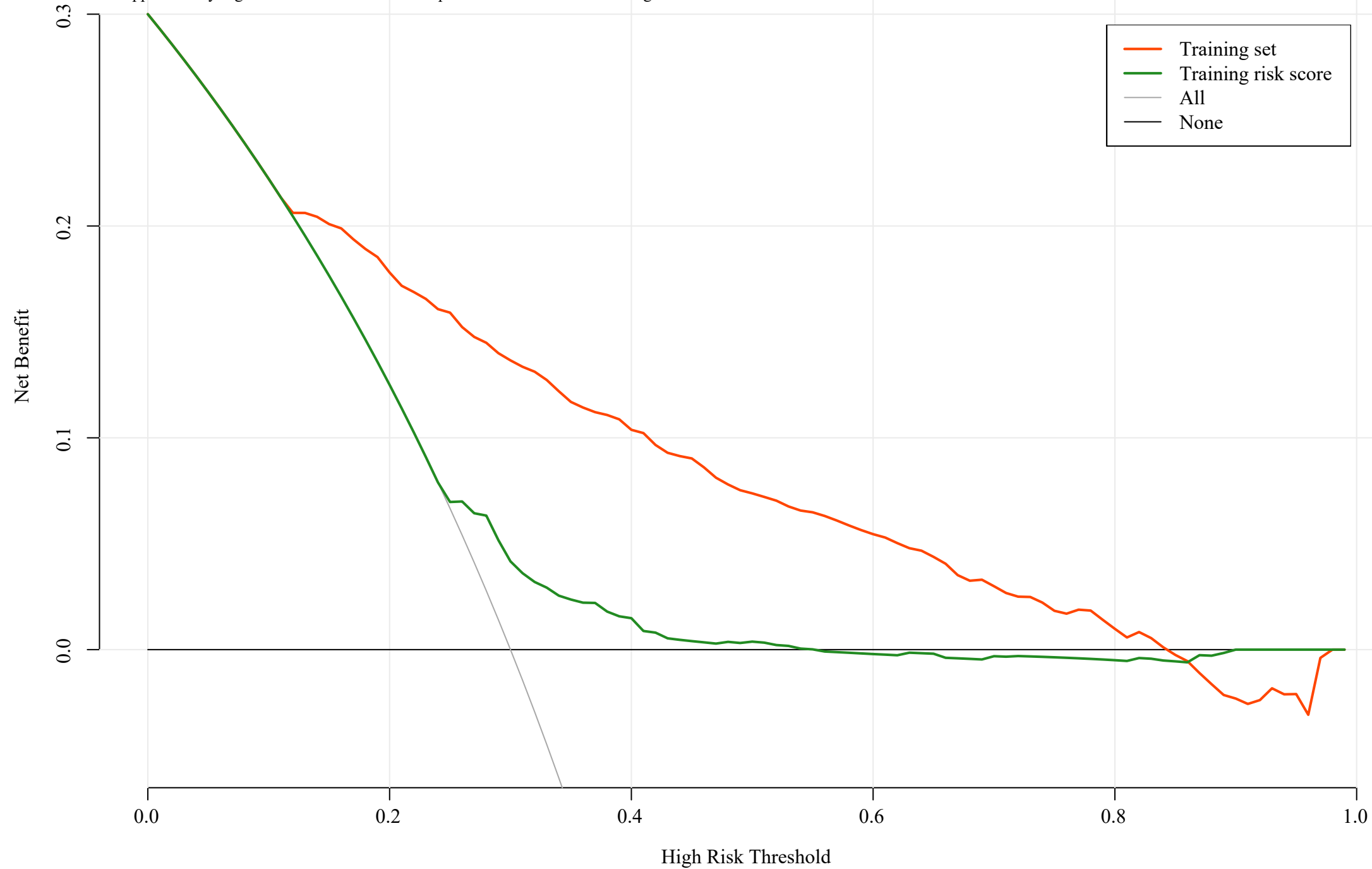

Supplement: Supplementary file 6 — Additional file 6: Supplementary Figure 5. The DCA curve of our prediction model in the training set. [file 12872_2023_3521_MOESM6_ESM.pdf]

Supplementary Figure 6 The DCA curve of our prediction model in the testing set.

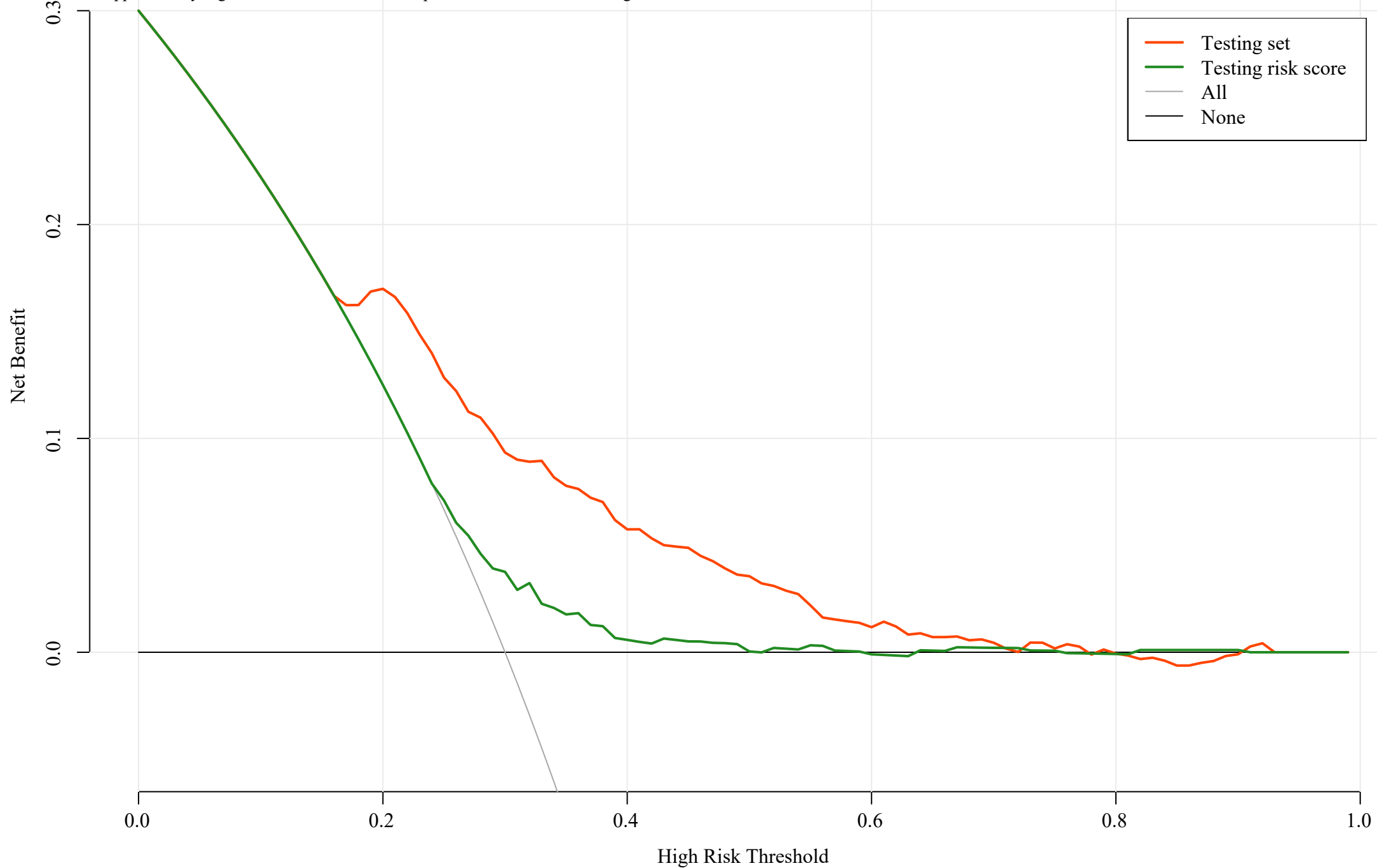

Supplement: Supplementary file 7 — Additional file 7: Supplementary Figure 6. The DCA curve of our prediction model in the testing set. [file 12872_2023_3521_MOESM7_ESM.pdf]

Supplementary Figure 7 The DCA curve of our prediction model in the internal validation set.

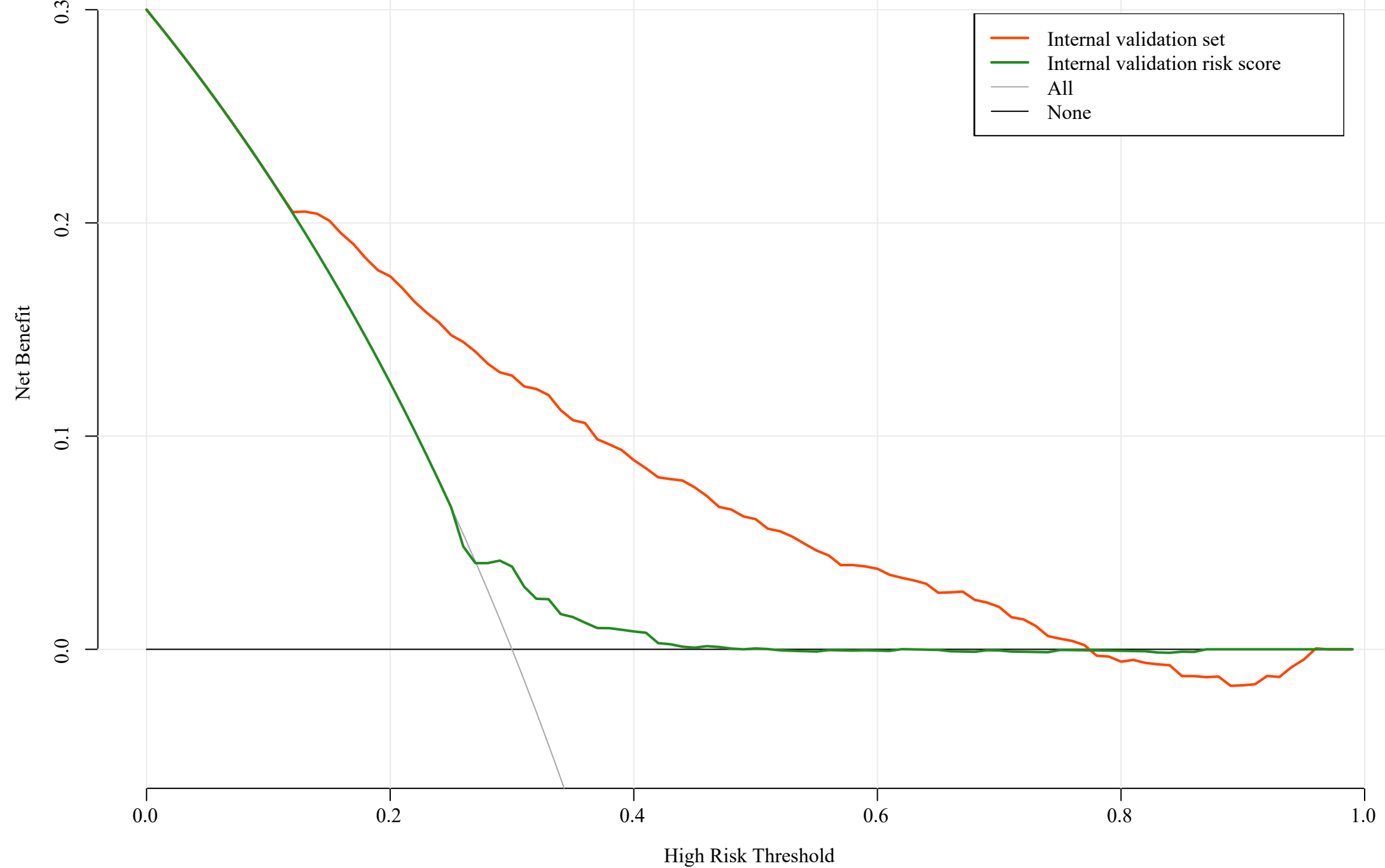

Supplement: Supplementary file 8 — Additional file 8: Supplementary Figure 7. The DCA curve of our prediction model in the internal validation set. [file 12872_2023_3521_MOESM8_ESM.pdf]

Supplementary Figure 8 The DCA curve of our prediction model in the external validation set.

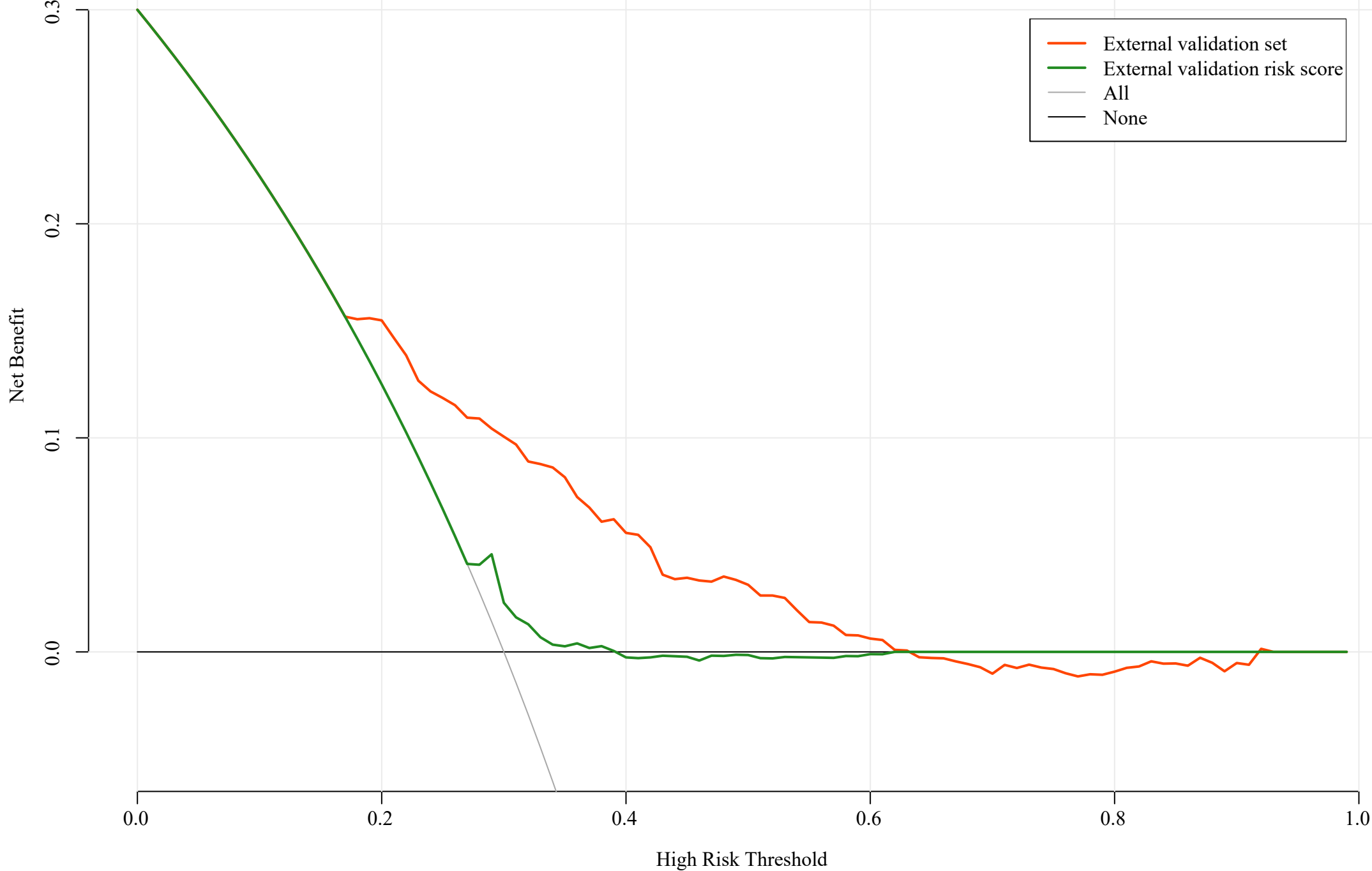

Supplement: Supplementary file 9 — Additional file 9: Supplementary Figure 8. The DCA curve of our prediction model in the external validation set. [file 12872_2023_3521_MOESM9_ESM.pdf]
